# Metabolic Alterations in *FMR1* Premutation Carriers

**DOI:** 10.3389/fmolb.2020.571092

**Published:** 2020-09-18

**Authors:** Yiqu Cao, Yun Peng, Ha Eun Kong, Emily G. Allen, Peng Jin

**Affiliations:** ^1^Department of Human Genetics, School of Medicine, Emory University, Atlanta, GA, United States; ^2^Department of Neurology, Xiangya Hospital, Central South University, Changsha, China

**Keywords:** FXTAS, *FMR1*, metabolomics, biomarker, therapeutic development

## Abstract

*FMR1* gene premutation carriers are at risk of developing Fragile X-associated tremor/ataxia syndrome (FXTAS) and Fragile X-associated primary ovarian insufficiency (FXPOI) in adulthood. Currently the development of biomarkers and effective treatments in *FMR1* premutations is still in its infancy. Recent metabolic studies have shown novel findings in asymptomatic *FMR1* premutation carriers and FXTAS, which provide promising insight through identification of potential biomarkers and therapeutic pathways. Here we review the latest advancements of the metabolic alterations found in asymptomatic *FMR1* premutation carriers and FXTAS, along with our perspective for future studies in this emerging field.

## Introduction

A 55–200 CGG repeat expansion in the 5′ UTR of the fragile X mental retardation 1 (*FMR1*) gene is the hallmark of premutation carriers. The *FMR1* premutation carriers are at risk of developing Fragile X-associated tremor/ataxia syndrome (FXTAS) and Fragile X-associated primary ovarian insufficiency (FXPOI) in adulthood (Allingham-Hawkins et al., 1999; Hagerman et al., 2001), as well as other unspecific syndromes (Mila et al., 2018). FXTAS is a neurodegenerative disorder predominantly in men, characterized by kinetic tremor, gait ataxia, parkinsonism, executive dysfunction, and neuropathy (Jacquemont et al., 2003; Hall and Berry-Kravis, 2018); and FXPOI is a condition in women characterized by reduced function of the ovaries (Man et al., 2017). In FXTAS, the two principal molecular mechanisms are: (1) RNA-gain-of function toxicity (Jin et al., 2003, 2007; Tassone et al., 2004; Sofola et al., 2007; Sellier et al., 2010), which leads to sequestration of various rCGG repeat-binding proteins; and (2) repeat-associated non-ATG (RAN) translation (Todd et al., 2013; Oh et al., 2015; Krans et al., 2016; Sellier et al., 2017), which produces the polyglycine (polyG) peptides toxic to cells. As another phenotype of *FMR1* premutation, FXPOI is believed to share similar molecular mechanisms with FXTAS based on current evidence, but still needs further research (Elizur et al., 2014, 2019; Buijsen et al., 2016; Man et al., 2017). Currently, there is no effective treatment for FXTAS and FXPOI, and the development of biomarkers is still in its infancy (Hagerman and Hagerman, 2016; Man et al., 2017).

Recently, several studies have made encouraging discoveries in the metabolomics of asymptomatic *FMR1* premutation carriers and FXTAS, which provide promising insight for the identification of potential biomarkers and therapeutic pathways. Currently, no metabolomic study in FXPOI has been reported. Therefore, in this review, we focus on the current knowledge of metabolic alterations in asymptomatic *FMR1* premutation carriers and FXTAS. We begin by describing the basic concepts of metabolism, then discuss the specific metabolic alterations associated with *FMR1* premutation carriers, and lastly provide an overview of future directions in this field.

## Metabolomics

Metabolomics explores the metabolic alterations associated with health and disease (Adamski, 2020). More specifically, metabolomics detects the metabolites and small molecular chemicals in various sample types, including biofluids, cells, and tissues (Johnson et al., 2016). The common human sample types used in the metabolomics include plasma, cerebrospinal fluid (CSF), peripheral blood mononuclear cells (PBMCs), fibroblasts and muscle tissues. A combination of analytical tools is implemented to detect various chemicals in the samples, such as liquid chromatography – mass spectrometry (LC-MS) and gas chromatography – mass spectrometry (GC–MS) (Wishart, 2019). The subsequent data analysis requires the available well-established databases (e.g., METLIN, HMDB, LipidMaps, and MassBank) (Fahy et al., 2009; Horai et al., 2010; Guijas et al., 2018; Wishart et al., 2018).

There are two main study types in metabolomics: untargeted (global) and targeted metabolomics (Johnson et al., 2016). Untargeted metabolomics detects the widest range of metabolites extracted from a sample, identifying novel and unanticipated alterations, whereas targeted metabolic analysis detects the levels of specific metabolites based on prior knowledge, allowing for higher sensitivity and selectivity (Johnson et al., 2016).

The main advantage of metabolomics is the ability to detect subtle perturbations in biological pathways. The metabolic signals could be amplified greatly because the most downstream changes of the genome, epigenome, transcriptome, and proteome are being measured (Urbanczyk-Wochniak et al., 2003). Based on this advantage, metabolic profiling has identified perturbations and novel biomarkers in many neurodegenerative diseases, such as Parkinson’s disease (PD) (Ahmed et al., 2009; Burte et al., 2017; Zhao et al., 2018), Alzheimer’s disease (AD) (Chang et al., 2015; Van Assche et al., 2015; Wu et al., 2016) and Huntington disease (HD) (Verwaest et al., 2011).

## Metabolic Alterations Associated With *FMR1* Premutation

### Carbohydrate Metabolism

Carbohydrate metabolism is fundamental for cellular energy balance and the biosynthesis of new cellular components (Dashty, 2013). In *FMR1* premutation carriers, researchers have identified alterations in carbohydrate metabolism pathways mainly in glycolysis, Krebs cycle, oxidative phosphorylation (OXPHOS), and the pentose phosphate shunt, as described below.

**FIGURE 1:**
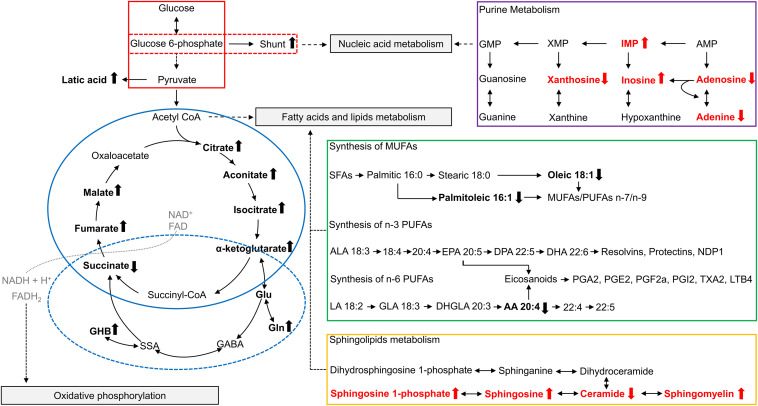
Overview of metabolites related to *FMR1* premutation. Altered metabolites identified in studies of plasma from *FMR1* premutation carriers are shown in bold and in black. Altered metabolites identified in studies of FXTAS mice cerebellum are show in bold and in red. The up arrow indicates the increase, and the down arrow indicates the decrease. The “shunt” refers to the pentose phosphate shunt. The glycolysis pathway is outlined in red, with the pentose phosphate shunt outlined in a dashed red box. The TCA cycle is outlined in solid blue, and GABA metabolism is outlined in a dashed blue circle. Purine metabolism is outlined in purple, synthesis of polyunsaturated fatty acids is outlined in green, and sphingolipid metabolism is outlined in orange. AA, arachidonic acid; ALA, α-linolenic acid; AMP, Adenosine monophosphate; CoA, Coenzyme A; DHA, docosahexaenoic acid; DHGLA, dihomo-γ-linolenic acid; DPA, docosapentaenoic acid; EPA, eicosapentaenoic acid; FAD, Flavin adenine dinucleotide; GABA, γ-Aminobutyric acid; GHB, 4-hydroxybutyrate; GLA, Gamma linolenic acid; Gln, Glutamine; Glu, Glutamate; GMP, Guanosine monophosphate; IMP, Inosine monophosphate; LA, linoleic acid; LTB4, Leukotriene B4; MUFAs, monounsaturated fatty acids; NAD+, Nicotinamide adenine dinucleotide; PGA1, Prostaglandin A1; PGE2, Prostaglandin E2; PGF2a, Prostaglandin F2alpha; PGI2, prostaglandin I2; PUFAs, polyunsaturated fatty acids; SFAs, saturated fatty acids; SSA, succinic semialdehyde; TXA2, Thromboxane A2; XMP, Xanthosine monophosphate.

Glycolysis is the catabolic pathway that converts glucose into pyruvate, serving as the common initiation pathway of anaerobic and aerobic oxidation of glucose (Figure 1, red box). Studies have reported changes in the intermediates and products of glycolysis in *FMR1* premutation carriers. In the plasma of premutation carriers, the levels of pyruvate entering the Krebs cycle were diminished due to the inhibition of the pyruvate dehydrogenase complex (PDHC), which may be caused by a higher [NADH]/[NAD+] ratio (Giulivi et al., 2016b). In addition to PDHC, high [NADH]/[NAD+] ratios could also inhibit other NAD-dependent dehydrogenases in the Krebs cycle, such as α-ketoglutarate dehydrogenase (AKGDH) and isocitrate dehydrogenase (ICDH). Furthermore, the lower entry of pyruvate into the Krebs cycle resulted in higher lactate formation in premutation carriers (Giulivi et al., 2016b).

In another study, peripheral blood mononuclear cells (PBMCs) were used to investigate mitochondrial energy-providing systems, and a dynamic alteration of glycolysis was observed when comparing controls, FXTAS-free carriers and FXTAS-affected carriers (Napoli et al., 2016). Specifically, FXTAS-free carriers exhibited a comparable or higher abundance of glycolytic proteins (e.g., hexokinase, phosphofructokinase, and pyruvate kinase) than controls, indicating an up-regulation of glycolysis (Napoli et al., 2016). However, in FXTAS-affected carriers, almost all glycolytic proteins were lower compared to control cells, with the exception of glucose-6-phosphate-dehydrogenase, which was higher in FXTAS-affected carriers compared to both controls and FXTAS-free carriers (Napoli et al., 2016). This marked alteration in glycolysis in FXTAS-affected carriers may implicate glycolysis in FXTAS pathogenesis (Napoli et al., 2016).

The Krebs cycle [also known as citric acid cycle (CAC) or tricarboxylic acid cycle (TCA cycle)] is a series of cyclic reactions that begins with the oxidation of acetyl-CoA into carbon dioxide and adenosine triphosphate (ATP), serving as the common metabolic pathway of carbohydrate, lipid and protein oxygenolysis (Figure 1, blue circle). Several intermediates of the Krebs cycle, located in the first half of the cycle, namely citrate, isocitrate and aconitate, were increased in the plasma of carriers compared to controls, indicating a decreased Krebs cycle activity in premutation (Giulivi et al., 2016a,b). Consistently, most enzymes of the Krebs cycle also showed a lower abundance in the PBMCs of the FXTAS-affected compared to controls (Napoli et al., 2016).

Oxidative Phosphorylation (OXPHOS) is the metabolic pathway in which cells utilize a series of protein complexes to produce ATP (Schmidt-Rohr, 2020). The activities of OXPHOS protein complexes and citrate synthase were lower in the PBMCs of carriers than controls, consistent with the relatively lower overall OXPHOS capacity in premutation carriers (Napoli et al., 2016). The combination of decreased OXPHOS and increased glycolysis in FXTAS-free premutation carriers suggests a Warburg effect in which glucose is mainly oxidized to lactate rather than undergoing OXPHOS to produce ATP (Warburg, 1956; Lin et al., 2012). These findings were accompanied by the observation of higher production of reactive oxygen species (ROS) and proton leak, as well as other mitochondrial outcomes (impaired Complex I activity, impaired redox-regulated mitochondrial disulfide relay system and increased mtDNA deletions) in the PBMCs or fibroblasts of premutation carriers, together suggesting increased oxidative-nitrative damage (Napoli et al., 2016; Song et al., 2016). Nitrative damage of scaffolding proteins could alter cytoskeletal organization, cause mitochondrial damage, and affect neuron maintenance and remodeling (Song et al., 2016).

The pentose phosphate shunt is a metabolic pathway that utilizes glucose-6-phosphate to generate NADPH and ribose 5-phosphate (Figure 1, red dashed box). As mentioned above, most glycolytic proteins in PBMCs were diminished in FXTAS-affected carriers compared to controls, except glucose-6-phosphate dehydrogenase, which is the pentose phosphate shunt entry point (Napoli et al., 2016). The higher abundance of glucose-6-phosphate-dehydrogenase in FXTAS-affected carriers indicates a shift of glucose toward the pentose phosphate shunt, probably aiming to produce NADPH, reducing equivalents required for the antioxidant defenses (Napoli et al., 2016).

### Amino Acids, Derivatives, and Biogenic Amines

The metabolism of amino acids, derivatives and biogenic amines has a profound effect on the nervous system by affecting the levels of structural proteins and neurotransmitters. In the plasma from premutation carriers, seventeen related metabolites (amino acids, derivatives or biogenic amines) were found to be altered, and among these, five amino acids or derivatives (proline, glycine, hydroxyproline, citrulline, and glutamylvaline) correlated with CGG repeat size, indicating a genotype-phenotype correlation (Giulivi et al., 2016b). Additionally, the increased plasma levels of glutamate and 4-hydroxybutyrate (GHB) may signal an imbalance in neurotransmission, which has been reported as a hallmark of anxiety disorders (Giulivi et al., 2016b). This observation is consistent with a higher incidence of anxiety/mood disorders in FXTAS-affected carriers (Bourgeois et al., 2007; Kogan et al., 2008).

Moreover, Giulivi et al. (2016a) hypothesized that given the increased levels of the Krebs cycle intermediates (citrate, isocitrate and aconitate), the activity of AKGDH is decreased in premutation carriers, resulting in an increased flux from alpha-ketoglutarate to glutamate, which subsequently culminates in the elevation of glutamine, GABA, and GHB levels (Figure 1, blue dashed circle). Consistently, researchers found that the metabotropic glutamate (Glu) receptor 5 and GABA pathways are altered in the brains of FXTAS patients (Pretto et al., 2014). Additionally, lower plasma concentrations of phenylethylamine (PEA) were also found in premutation carriers, which may reflect incipient nigrostriatal degeneration (Giulivi et al., 2016a).

In the cerebellum of FXTAS mice expressing r(CGG)_90_ in Purkinje cells, our group also observed alterations of amino acids (41 out of 115) (Kong et al., 2019). For example, 5-oxoproline was decreased in aged FXTAS compared to aged WT mice, suggesting that this metabolite is altered due to CGG-associated toxicity. 5-oxoproline was also decreased in the aged FXTAS compared to young FXTAS mice, implicating perturbation of this metabolite in the progression of FXTAS. Interestingly, subsequent genetic screening using a *Drosophila* model of FXTAS revealed that knockdown of *CG4306*, the fly ortholog of *Gamma-glutamylcyclotransferase (Ggct)*, which encodes the enzyme responsible for catalyzing the formation of 5-oxoproline (Oakley et al., 2008), resulted in suppression of (CGG)_90_ toxicity in *Drosophila*. This finding suggests *Ggct* as a genetic modifier of CGG-associated neurotoxicity (Kong et al., 2019), providing a novel therapeutic target for FXTAS.

### Fatty Acids and Structural Lipids

Giulivi et al. (2016b) found overall fatty acids levels decreased in the plasma of premutation carriers. More specifically, they found lower plasma levels of free fatty acids, oleic and arachidonic acids, which are associated with depression and parkinsonism (Giulivi et al., 2016b). Additionally, they found a decreased ratio of polyunsaturated fatty acids (PUFA) of the n-3 series over that of the n-6 series, which might be related to impaired learning and memory in premutation carriers (Figure 1, green box) (Giulivi et al., 2016b). The decreased ratio of the n-3 series over the n-6 series could result in more pro-inflammatory prostaglandins produced via the Δ5–6 desaturase pathway, and an increased pro-inflammatory status (Giulivi et al., 2016b). Moreover, researchers observed a decrease in palmitoleic acid in plasma from premutation carriers, along with the lower estimated enzymatic activity of stearoyl-CoA desaturase 1 (SCD1), the rate-limiting enzyme in monounsaturated fatty acid biosynthesis (Ntambi and Miyazaki, 2004), which is necessary for axonogenesis, neuron differentiation and carbohydrate utilization in brain (Giulivi et al., 2016b).

Fatty acids and their derivatives also have an essential role in maintaining cellular integrity as structural lipids. Sphingolipids are structural lipids for eukaryotic cell membranes, consisting of the sphingoid backbone, which is N-acylated with various fatty acids to form ceramide species (Maceyka and Spiegel, 2014). Recently, sphingolipid metabolism was found to be altered in the cerebellum of FXTAS mice (Figure 1, orange box) (Kong et al., 2019). Specifically, levels of sphingosine, sphingosine 1-phosphate, and sphingomyelin were increased, while levels of ceramide were decreased in FXTAS mice compared to wildtype (Kong et al., 2019). Further pathway analysis and subsequent validation in a FXTAS *Drosophila* model confirmed two genes related to sphingosine metabolism, *Schlank* and *Sk2* (Kong et al., 2019). *Schlank* is the *Drosophila* ortholog of ceramide synthase, the enzyme synthesizing ceramide from sphingosine. *Sk2* is the *Drosophila* ortholog of sphingosine kinase responsible for phosphorylating sphingosine to yield S1P. Knockdown of *Schlank* and *Sk2* resulted in enhancement of premutation CGG repeat-mediated neurodegeneration in *Drosophila*, indicating that *Schlank* and *Sk2* interact with the CGG repeat of *FMR1* and act as genetic modifiers in the neurodegeneration of FXTAS (Kong et al., 2019). The above findings support sphingolipid metabolism as a potential path for therapeutic development, and further research in human premutation carriers are warranted.

### Nucleotide Metabolism

In the same study (Kong et al., 2019), researchers found purine metabolism perturbed in the cerebellum of FXTAS mice, including increased inosine monophosphate (IMP) and inosine, and decreased xanthosine, adenosine, and adenine (Figure 1, purple box). Inosine 5′monophosphate dehydrogenase (Impdh) is the rate-limiting enzyme in guanine nucleotide biosynthesis, which catalyzes the conversion of IMP into xanthosine monophosphate (XMP). Knockdown of *Ras*, the fly ortholog of *Inosine Monophosphate Dehydrogenase 1 (Impdh1)*, which encodes Impdh, resulted in the enhancement of premutation CGG repeat-mediated neurodegeneration in the FXTAS *Drosophila* model (Kong et al., 2019). Consistently, disruptions in purine metabolism were also reported in the plasma from premutation carriers (Giulivi et al., 2016b). Imbalances in purine synthesis could affect multiple pathways including replication, transcription, and DNA repair, which may contribute to neurodegeneration in *FMR1* premutation carriers (Giulivi et al., 2016b; Kong et al., 2019).

## Therapeutic Potential Targeting Metabolic Pathways

Several recent studies have highlighted the breadth of metabolic alterations in the pathogenesis of *FMR1* premutation. As a result, targeting these perturbed metabolic pathways is expected to be a promising new strategy in the treatment of *FMR1* premutation carriers.

Consistent with this expectation, researchers have identified several antioxidants, including scavengers of superoxide, hydrogen peroxide and hydroxyl radicals, which could recover citrate synthase activity and mitochondrial function in fibroblasts from *FMR1* premutation carriers (Song et al., 2016). In a 12-week intervention study, Napoli et al. found that allopregnanolone treatment improved cognition and memory of FXTAS patients (Napoli et al., 2019). Plasma metabolomic profiling of FXTAS patients showed an improved value of glutamate/glutamine, a trend toward higher contents of succinate, and a decreased GHB concentration after allopregnanolone treatment, suggesting improvements in the activity of the succinic semialdehyde dehydrogenase (SSADH) mediated pathway (Napoli et al., 2019). The researchers hypothesized that the neuroprotective effect of allopregnolone in FXTAS may be due to the reduction of excessive GHB. GHB is a neuropharmacologically active compound, and excessive GHB could be neurotoxic as an inhibitor of presynaptic dopamine release (Bernasconi et al., 1999; Maitre et al., 2000). Overall, this study was a step forward in exploring potential drugs by targeting the metabolic pathways altered in *FMR1* premutation carriers. However, given the small sample size of this study, replication studies in larger cohorts are needed to confirm these findings. In addition, future therapy studies are encouraged to test the other metabolic pathways identified in *FMR1* premutation.

## Conclusion and Future Directions

Diverse metabolic alterations have been found in *FMR1* premutation carriers, including perturbations in the metabolism of carbohydrates, amino acids and derivatives, biogenic amines, fatty acids, structural lipids, and nucleotide. Of note, many of those metabolic alterations can be attributed to mitochondrial dysfunction, such as decreased OXPHOS capacity, changed mitochondrial proteins and altered mitochondrial architecture, which are caused by RNA gain-of function toxicity and RAN translation (Hukema et al., 2014; Alvarez-Mora et al., 2017; Drozd et al., 2019; Gohel et al., 2019; Nobile et al., 2020). As the most downstream of *FMR1* premutation pathogenesis, the metabolic alterations discussed in this review could provide resources in the search for biomarkers and promising therapeutic targets for *FMR1* premutation carriers. Additionally, although current findings are based on the asymptomatic *FMR1* premutation and FXTAS, these findings may also benefit future studies in FXPOI as they share the same genetic mechanism – the *FMR1* premutation.

Moving forward, we suggest taking the following into consideration. First, using a disease model system has the advantage of being able to assess the metabolic alterations specifically in the affected tissues, such as the cerebellum in FXTAS. However, these results must be correlated with findings in human studies for validation. Second, for the human studies, using a blood sample would be convenient, but the metabolism as measured in blood may not accurately reflect the metabolome of the specific affected tissues. For example, the degree to which plasma metabolomes reflect central nervous system (CNS) neurobiology remains uncertain due to the limitation of the blood-brain barrier. Therefore, an important step to take in future studies would be to use the cerebrospinal fluid (CSF), or brain organoids derived from induced pluripotent cells (iPSC), which may more accurately reflect the metabolome in CNS. Additionally, it would be valuable to correlate the peripheral metabolic alteration with neuroimaging, such as Magnetic Resonance Spectroscopy (MRS). Third, the sample sizes in existing human studies are relatively small. It is generally challenging to enroll a large sample of patients in the study of rare diseases such as *FMR1* premutation. Thus, future efforts are encouraged to overcome this limitation by increasing the number of multi-center studies.

## Author Contributions

YC and YP wrote the manuscript. HK, EA, and PJ edited the manuscript. All authors contributed to the article and approved the submitted version.

## Conflict of Interest

The authors declare that the research was conducted in the absence of any commercial or financial relationships that could be construed as a potential conflict of interest.

## References

[B1] Adamski J. (2020). “Chapter 1 – Introduction to metabolomics,” in *Metabolomics for Biomedical Research*, ed. Adamski J. (Cambridge, MA: Academic Press), 1–15. 10.1039/9781788019880-00001

[B2] Ahmed S. S. Santosh W. Kumar S. Christlet H. T. (2009). Metabolic profiling of Parkinson’s disease: evidence of biomarker from gene expression analysis and rapid neural network detection. *J. Biomed. Sci.* 16:63. 10.1186/1423-0127-16-63 19594911 PMC2720938

[B3] Allingham-Hawkins D. J. Babul-Hirji R. Chitayat D. Holden J. J. Yang K. T. Lee C. (1999). Fragile X premutation is a significant risk factor for premature ovarian failure: the International Collaborative POF in Fragile X study–preliminary data. *Am. J. Med. Genet.* 83 322–325. 10.1002/(sici)1096-8628(19990402)83:4<322::aid-ajmg17>3.0.co;2-b 10208170 PMC3728646

[B4] Alvarez-Mora M. I. Rodriguez-Revenga L. Madrigal I. Guitart-Mampel M. Garrabou G. Mila M. (2017). Impaired mitochondrial function and dynamics in the pathogenesis of FXTAS. *Mol. Neurobiol.* 54 6896–6902. 10.1007/s12035-016-0194-7 27771901

[B5] Bernasconi R. Mathivet P. Bischoff S. Marescaux C. (1999). Gamma-hydroxybutyric acid: an endogenous neuromodulator with abuse potential? *Trends Pharmacol. Sci.* 20 135–141. 10.1016/s0165-6147(99)01341-3 10322498

[B6] Bourgeois J. A. Cogswell J. B. Hessl D. Zhang L. Ono M. Y. Tassone F. (2007). Cognitive, anxiety and mood disorders in the fragile X-associated tremor/ataxia syndrome. *Gen. Hosp. Psychiatry* 29 349–356. 10.1016/j.genhosppsych.2007.03.003 17591512 PMC3991490

[B7] Buijsen R. A. Visser J. A. Kramer P. Severijnen E. A. Gearing M. Charlet-Berguerand N. (2016). Presence of inclusions positive for polyglycine containing protein, FMRpolyG, indicates that repeat-associated non-AUG translation plays a role in fragile X-associated primary ovarian insufficiency. *Hum. Reprod.* 31 158–168. 10.1093/humrep/dev280 26537920 PMC4677964

[B8] Burte F. Houghton D. Lowes H. Pyle A. Nesbitt S. Yarnall A. (2017). metabolic profiling of Parkinson’s disease and mild cognitive impairment. *Mov. Disord.* 32 927–932. 28394042 10.1002/mds.26992 PMC5485028

[B9] Cai R. Zhang Y. Simmering J. E. Schultz J. L. Li Y. Fernandez-Carasa I. (2019). Enhancing glycolysis attenuates Parkinson’s disease progression in models and clinical databases. *J. Clin. Invest.* 129 4539–4549. 10.1172/jci129987 31524631 PMC6763248

[B10] Chang K. L. Pee H. N. Tan W. P. Dawe G. S. Holmes E. Nicholson J. K. (2015). Metabolic profiling of CHO-AbetaPP695 cells revealed mitochondrial dysfunction prior to amyloid-beta pathology and potential therapeutic effects of both PPARgamma and PPARalpha Agonisms for Alzheimer’s disease. *J. Alzheimers Dis.* 44 215–231. 10.3233/jad-140429 25201780

[B11] Chaudhuri A. D. Kabaria S. Choi D. C. Mouradian M. M. Junn E. (2015). MicroRNA-7 promotes glycolysis to protect against 1-Methyl-4-phenylpyridinium-induced cell death. *J. Biol. Chem.* 290 12425–12434. 10.1074/jbc.m114.625962 25814668 PMC4424371

[B12] Dashty M. (2013). A quick look at biochemistry: carbohydrate metabolism. *Clin. Biochem.* 46 1339–1352. 10.1016/j.clinbiochem.2013.04.027 23680095

[B13] Drozd M. Delhaye S. Maurin T. Castagnola S. Grossi M. Brau F. (2019). Reduction of Fmr1 mRNA levels rescues pathological features in cortical neurons in a model of FXTAS. *Mol. Ther. Nucleic Acids* 18 546–553. 10.1016/j.omtn.2019.09.018 31671347 PMC6838541

[B14] Elizur S. E. Friedman Gohas M. Dratviman-Storobinsky O. Cohen Y. (2019). Pathophysiology mechanisms in Fragile-X primary ovarian insufficiency. *Methods Mol. Biol.* 1942 165–171. 10.1007/978-1-4939-9080-1_14 30900184

[B15] Elizur S. E. Lebovitz O. Derech-Haim S. Dratviman-Storobinsky O. Feldman B. Dor J. (2014). Elevated levels of FMR1 mRNA in granulosa cells are associated with low ovarian reserve in FMR1 premutation carriers. *PLoS One* 9:e105121. 10.1371/journal.pone.0105121 25153074 PMC4143194

[B16] Fahy E. Subramaniam S. Murphy R. C. Nishijima M. Raetz C. R. Shimizu T. (2009). Update of the LIPID MAPS comprehensive classification system for lipids. *J. Lipid Res.* 50 S9–S14. 19098281 10.1194/jlr.R800095-JLR200 PMC2674711

[B17] Giulivi C. Napoli E. Tassone F. Halmai J. Hagerman R. (2016a). Plasma biomarkers for monitoring brain pathophysiology in FMR1 premutation carriers. *Front. Mol. Neurosci.* 9:71. 10.3389/fnmol.2016.00071 27570505 PMC4981605

[B18] Giulivi C. Napoli E. Tassone F. Halmai J. Hagerman R. (2016b). Plasma metabolic profile delineates roles for neurodegeneration, pro-inflammatory damage and mitochondrial dysfunction in the FMR1 premutation. *Biochem. J.* 473 3871–3888. 10.1042/bcj20160585 27555610 PMC7014977

[B19] Gohel D. Sripada L. Prajapati P. Singh K. Roy M. Kotadia D. (2019). FMRpolyG alters mitochondrial transcripts level and respiratory chain complex assembly in Fragile X associated tremor/ataxia syndrome [FXTAS]. *Biochim. Biophys. Acta Mol. Basis Dis.* 1865 1379–1388. 10.1016/j.bbadis.2019.02.010 30771487

[B20] Gonzalez-Cabo P. Ros S. Palau F. (2010). Flavin adenine dinucleotide rescues the phenotype of frataxin deficiency. *PLoS One* 5:e8872. 10.1371/journal.pone.0008872 20111601 PMC2810331

[B21] Guijas C. Montenegro-Burke J. R. Domingo-Almenara X. Palermo A. Warth B. Hermann G. (2018). METLIN: a technology platform for identifying knowns and unknowns. *Anal. Chem.* 90 3156–3164. 10.1021/acs.analchem.7b04424 29381867 PMC5933435

[B22] Hagerman R. J. Hagerman P. (2016). Fragile X-associated tremor/ataxia syndrome – features, mechanisms and management. *Nat. Rev. Neurol.* 12 403–412. 10.1038/nrneurol.2016.82 27340021

[B23] Hagerman R. J. Leehey M. Heinrichs W. Tassone F. Wilson R. Hills J. (2001). Intention tremor, parkinsonism, and generalized brain atrophy in male carriers of fragile X. *Neurology* 57 127–130. 10.1212/wnl.57.1.127 11445641

[B24] Hall D. A. Berry-Kravis E. (2018). Fragile X syndrome and fragile X-associated tremor ataxia syndrome. *Handb. Clin. Neurol.* 147 377–391. 29325626 10.1016/B978-0-444-63233-3.00025-7

[B25] Horai H. Arita M. Kanaya S. Nihei Y. Ikeda T. Suwa K. (2010). MassBank: a public repository for sharing mass spectral data for life sciences. *J. Mass Spectrom* 45 703–714. 10.1002/jms.1777 20623627

[B26] Hukema R. K. Buijsen R. A. Raske C. Severijnen L. A. Nieuwenhuizen-Bakker I. Minneboo M. (2014). Induced expression of expanded CGG RNA causes mitochondrial dysfunction in vivo. *Cell Cycle* 13 2600–2608. 10.4161/15384101.2014.943112 25486200 PMC4614669

[B27] Jacquemont S. Hagerman R. J. Leehey M. Grigsby J. Zhang L. Brunberg J. A. (2003). Fragile X premutation tremor/ataxia syndrome: molecular, clinical, and neuroimaging correlates. *Am. J. Hum. Genet.* 72 869–878. 12638084 10.1086/374321 PMC1180350

[B28] Jin P. Duan R. Qurashi A. Qin Y. Tian D. Rosser T. C. (2007). Pur alpha binds to rCGG repeats and modulates repeat-mediated neurodegeneration in a Drosophila model of fragile X tremor/ataxia syndrome. *Neuron* 55 556–564. 10.1016/j.neuron.2007.07.020 17698009 PMC1994817

[B29] Jin P. Zarnescu D. C. Zhang F. Pearson C. E. Lucchesi J. C. Moses K. (2003). RNA-mediated neurodegeneration caused by the fragile X premutation rCGG repeats in Drosophila. *Neuron* 39 739–747. 10.1016/s0896-6273(03)00533-6 12948442

[B30] Johnson C. H. Ivanisevic J. Siuzdak G. (2016). Metabolomics: beyond biomarkers and towards mechanisms. *Nat. Rev. Mol. Cell Biol.* 17 451–459. 10.1038/nrm.2016.25 26979502 PMC5729912

[B31] Kogan C. S. Turk J. Hagerman R. J. Cornish K. M. (2008). Impact of the Fragile X mental retardation 1 (FMR1) gene premutation on neuropsychiatric functioning in adult males without fragile X-associated Tremor/Ataxia syndrome: a controlled study. *Am. J. Med. Genet. B Neuropsychiatr. Genet.* 147B 859–872. 10.1002/ajmg.b.30685 18165971

[B32] Kong H. E. Lim J. Zhang F. Huang L. Gu Y. Nelson D. L. (2019). Metabolic pathways modulate the neuronal toxicity associated with fragile X-associated tremor/ataxia syndrome. *Hum. Mol. Genet.* 28 980–991. 10.1093/hmg/ddy410 30476102 PMC6400045

[B33] Krans A. Kearse M. G. Todd P. K. (2016). Repeat-associated non-AUG translation from antisense CCG repeats in fragile X tremor/ataxia syndrome. *Ann. Neurol.* 80 871–881. 10.1002/ana.24800 27761921 PMC5177492

[B34] Lin C. C. Cheng T. L. Tsai W. H. Tsai H. J. Hu K. H. Chang H. C. (2012). Loss of the respiratory enzyme citrate synthase directly links the Warburg effect to tumor malignancy. *Sci. Rep.* 2:785. 10.1038/srep00785 PMC3492867 23139858

[B35] Maceyka M. Spiegel S. (2014). Sphingolipid metabolites in inflammatory disease. *Nature* 510 58–67. 10.1038/nature13475 24899305 PMC4320971

[B36] Maitre M. Andriamampandry C. Kemmel V. Schmidt C. Hode Y. Hechler V. (2000). Gamma-hydroxybutyric acid as a signaling molecule in brain. *Alcohol* 20 277–283. 10.1016/s0741-8329(99)00092-0 10869870

[B37] Man L. Lekovich J. Rosenwaks Z. Gerhardt J. (2017). Fragile X-associated diminished ovarian reserve and primary ovarian insufficiency from molecular mechanisms to clinical manifestations. *Front. Mol. Neurosci.* 10:290. 10.3389/fnmol.2017.00290 28955201 PMC5600956

[B38] Mila M. Alvarez-Mora M. I. Madrigal I. Rodriguez-Revenga L. (2018). Fragile X syndrome: an overview and update of the FMR1 gene. *Clin. Genet.* 93 197–205. 10.1111/cge.13075 28617938

[B39] Napoli E. Schneider A. Wang J. Y. Trivedi A. Carrillo N. R. Tassone F. (2019). Allopregnanolone treatment improves plasma metabolomic profile associated with GABA metabolism in Fragile X-associated tremor/ataxia syndrome: a pilot study. *Mol. Neurobiol.* 56 3702–3713. 10.1007/s12035-018-1330-3 30187385 PMC6401336

[B40] Napoli E. Song G. Schneider A. Hagerman R. Eldeeb M. A. Azarang A. (2016). Warburg effect linked to cognitive-executive deficits in FMR1 premutation. *FASEB J.* 30 3334–3351. 10.1096/fj.201600315r 27335370 PMC5024697

[B41] Nobile V. Palumbo F. Lanni S. Ghisio V. Vitali A. Castagnola M. (2020). Altered mitochondrial function in cells carrying a premutation or unmethylated full mutation of the FMR1 gene. *Hum. Genet.* 139 227–245. 10.1007/s00439-019-02104-7 31919630

[B42] Ntambi J. M. Miyazaki M. (2004). Regulation of stearoyl-CoA desaturases and role in metabolism. *Prog. Lipid Res.* 43 91–104. 10.1016/s0163-7827(03)00039-0 14654089

[B43] Oakley A. J. Yamada T. Liu D. Coggan M. Clark A. G. Board P. G. (2008). The identification and structural characterization of C7orf24 as gamma-glutamyl cyclotransferase. An essential enzyme in the gamma-glutamyl cycle. *J. Biol. Chem.* 283 22031–22042. 10.1074/jbc.m803623200 18515354

[B44] Oh S. Y. He F. Krans A. Frazer M. Taylor J. P. Paulson H. L. (2015). RAN translation at CGG repeats induces ubiquitin proteasome system impairment in models of fragile X-associated tremor ataxia syndrome. *Hum. Mol. Genet.* 24 4317–4326. 10.1093/hmg/ddv165 25954027 PMC4492395

[B45] Pretto D. I. Kumar M. Cao Z. Cunningham C. L. Durbin-Johnson B. Qi L. (2014). Reduced excitatory amino acid transporter 1 and metabotropic glutamate receptor 5 expression in the cerebellum of fragile X mental retardation gene 1 premutation carriers with fragile X-associated tremor/ataxia syndrome. *Neurobiol. Aging* 35 1189–1197. 10.1016/j.neurobiolaging.2013.11.009 24332449 PMC4062976

[B46] Schmidt-Rohr K. (2020). Oxygen is the high-energy molecule powering complex multicellular life: fundamental corrections to traditional bioenergetics. *ACS Omega* 5 2221–2233. 10.1021/acsomega.9b03352 32064383 PMC7016920

[B47] Sellier C. Buijsen R. A. M. He F. Natla S. Jung L. Tropel P. (2017). Translation of expanded CGG repeats into FMRpolyG is pathogenic and may contribute to Fragile X Tremor ataxia syndrome. *Neuron* 93 331–347. 10.1016/j.neuron.2016.12.016 28065649 PMC5263258

[B48] Sellier C. Rau F. Liu Y. Tassone F. Hukema R. K. Gattoni R. (2010). Sam68 sequestration and partial loss of function are associated with splicing alterations in FXTAS patients. *Embo J* 29 1248–1261. 10.1038/emboj.2010.21 20186122 PMC2857464

[B49] Sofola O. A. Jin P. Qin Y. Duan R. Liu H. De Haro M. (2007). RNA-binding proteins hnRNP A2/B1 and CUGBP1 suppress fragile X CGG premutation repeat-induced neurodegeneration in a Drosophila model of FXTAS. *Neuron* 55 565–571. 10.1016/j.neuron.2007.07.021 17698010 PMC2215388

[B50] Song G. Napoli E. Wong S. Hagerman R. Liu S. Tassone F. (2016). Altered redox mitochondrial biology in the neurodegenerative disorder fragile X-tremor/ataxia syndrome: use of antioxidants in precision medicine. *Mol. Med.* 22 548–559. 10.2119/molmed.2016.00122 27385396 PMC5082295

[B51] Tassone F. Iwahashi C. Hagerman P. J. (2004). FMR1 RNA within the intranuclear inclusions of fragile X-associated tremor/ataxia syndrome (FXTAS). *RNA Biol.* 1 103–105. 10.4161/rna.1.2.1035 17179750

[B52] Teo E. Ravi S. Barardo D. Kim H. S. Fong S. Cazenave-Gassiot A. (2019). Metabolic stress is a primary pathogenic event in transgenic Caenorhabditis elegans expressing pan-neuronal human amyloid beta. *eLife* 8:e50069. 10.7554/eLife.50069 PMC6794093 31610847

[B53] Todd P. K. Oh S. Y. Krans A. He F. Sellier C. Frazer M. (2013). CGG repeat-associated translation mediates neurodegeneration in fragile X tremor ataxia syndrome. *Neuron* 78 440–455. 10.1016/j.neuron.2013.03.026 23602499 PMC3831531

[B54] Urbanczyk-Wochniak E. Luedemann A. Kopka J. Selbig J. Roessner-Tunali U. Willmitzer L. (2003). Parallel analysis of transcript and metabolic profiles: a new approach in systems biology. *EMBO Rep* 4, 989–993. 10.1038/sj.embor.embor944 12973302 PMC1326402

[B55] Van Assche R. Temmerman L. Dias D. A. Boughton B. Boonen K. Braeckman B. P. (2015). Metabolic profiling of a transgenic *Caenorhabditis elegans* Alzheimer model. *Metabolomics* 11 477–486. 10.1007/s11306-014-0711-5 25750603 PMC4342517

[B56] Verdin E. (2015). NAD(+) in aging, metabolism, and neurodegeneration. *Science* 350 1208–1213. 10.1126/science.aac4854 26785480

[B57] Verwaest K. A. Vu T. N. Laukens K. Clemens L. E. Nguyen H. P. Van Gasse B. (2011). (1)H NMR based metabolomics of CSF and blood serum: a metabolic profile for a transgenic rat model of Huntington disease. *Biochim. Biophys. Acta* 1812 1371–1379. 10.1016/j.bbadis.2011.08.001 21867751

[B58] Wang X. Zhang R. Lin Y. Shi P. (2020). Inhibition of NF-kappaB might enhance the protective role of roflupram on SH-SY5Y cells under amyloid beta stimulation via PI3K/AKT/mTOR signaling pathway. *Int. J. Neurosci.* 10.1080/00207454.2020.1759588 [Epub ahead of print]. 32314929

[B59] Warburg O. (1956). On respiratory impairment in cancer cells. *Science* 124 269–270. 13351639

[B60] Wishart D. S. (2019). Metabolomics for investigating physiological and pathophysiological processes. *Physiol. Rev.* 99 1819–1875. 10.1152/physrev.00035.2018 31434538

[B61] Wishart D. S. Feunang Y. D. Marcu A. Guo A. C. Liang K. Vazquez-Fresno R. (2018). HMDB 4.0: the human metabolome database for 2018. *Nucleic Acids Res.* 46 D608–D617. 29140435 10.1093/nar/gkx1089 PMC5753273

[B62] Wu J. Fu B. Lei H. Tang H. Wang Y. (2016). Gender differences of peripheral plasma and liver metabolic profiling in APP/PS1 transgenic AD mice. *Neuroscience* 332 160–169. 10.1016/j.neuroscience.2016.06.049 27393253

[B63] Yao J. Brinton R. D. (2012). Estrogen regulation of mitochondrial bioenergetics: implications for prevention of Alzheimer’s disease. *Adv. Pharmacol.* 64 327–371. 10.1016/b978-0-12-394816-8.00010-6 22840752 PMC3970844

[B64] Zhao H. Wang C. Zhao N. Li W. Yang Z. Liu X. (2018). Potential biomarkers of Parkinson’s disease revealed by plasma metabolic profiling. *J. Chromatogr. B Anal. Technol. Biomed. Life Sci.* 1081-1082 101–108. 10.1016/j.jchromb.2018.01.025 29518718

